# Intermolecular Diels-Alder Cycloadditions of Furfural-Based Chemicals from Renewable Resources: A Focus on the Regio- and Diastereoselectivity in the Reaction with Alkenes

**DOI:** 10.3390/ijms222111856

**Published:** 2021-11-01

**Authors:** Konstantin I. Galkin, Valentine P. Ananikov

**Affiliations:** 1 Zelinsky Institute of Organic Chemistry, Russian Academy of Sciences, Leninsky Prospekt 47, 119991 Moscow, Russia; glkn@ioc.ac.ru; 2 Laboratory of Functional Composite Materials, Bauman Moscow State Technical University, 2nd Baumanskaya Street 5/1, 105005 Moscow, Russia

**Keywords:** biobased furans, renewable building blocks, plant biomass, Diels-Alder cycloaddition, selectivity, sustainable chemistry, biorefining

## Abstract

A recent strong trend toward green and sustainable chemistry has promoted the intensive use of renewable carbon sources for the production of polymers, biofuels, chemicals, monomers and other valuable products. The Diels-Alder reaction is of great importance in the chemistry of renewable resources and provides an atom-economic pathway for fine chemical synthesis and for the production of materials. The biobased furans furfural and 5-(hydroxymethyl)furfural, which can be easily obtained from the carbohydrate part of plant biomass, were recognized as “platform chemicals” that will help to replace the existing oil-based refining to biorefining. Diels-Alder cycloaddition of furanic dienes with various dienophiles represents the ideal example of a “green” process characterized by a 100% atom economy and a reasonable E-factor. In this review, we first summarize the literature data on the regio- and diastereoselectivity of intermolecular Diels-Alder reactions of furfural derivatives with alkenes with the aim of establishing the current progress in the efficient production of practically important low-molecular-weight products. The information provided here will be useful and relevant to scientists in many fields, including medical and pharmaceutical research, polymer development and materials science.

## 1. Introduction

To date, the development of efficient technologies for catalytic or biocatalytic conversion of renewable plant biomass into viable targeted products remains one of the most important and challenging tasks for modern chemical science [1,2,3,4,5]. The primary advantage of biorefining based on renewable carbon sources over traditional refining using exhaustible resources is the realization of a carbon-neutral cycle, leading to zero total carbon emissions into the environment during chemical production and consumption. Biobased furans—furfural (FF) and 5-(hydroxymethyl)furfural (HMF)—can be obtained by acid-catalyzed dehydration of carbohydrates and are recognized as “platform chemicals”. As expected, the key role of biobased technologies is to replace the key existing products of oil-based refinement with renewables [4,6,7]. The tremendous synthetic potential explains the unprecedented scale of research in the fields of synthesis and application of furanic platform chemicals for the production of biofuels, chemicals, polymers and other industrially important products, which was evidenced by the increasing number of relevant publications (partially since 2010, Figure 1) and was highlighted in many recent reviews [7,8,9,10,11,12,13,14,15,16,17,18,19,20].

**Figure 2:**
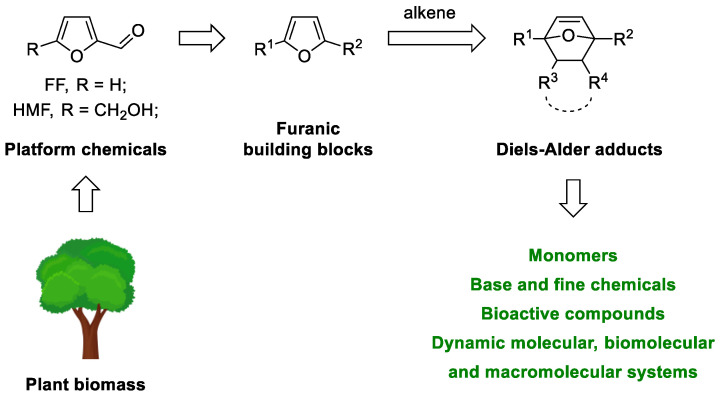
Diels-Alder cycloaddition with biobased furans as an approach towards practically important products. Summarizing and analyzing scientific data about the regio- and diastereoselectivity of intermolecular Diels-Alder cycloadditions between furfural derivatives and alkenes was a general aim of this review.

One of the focused reactions of furan chemistry is the [4+2]-cycloaddition, well known as the Diels-Alder (DA) reaction, in the classic mechanism based on the interaction of the highest occupied molecular orbital of furanic diene (HOMO_diene_) and the lowest unoccupied molecular orbital of dienophile (LUMO_dienophile_). The DA reaction may proceed with high efficiency under solvent-free and/or noncatalytic conditions, representing the ideal example of a “green” process characterized by a 100% atom economy and a low to moderate E-factor [21,22]. Intermolecular furan/alkene DA reactions have a high potential for application in fine organic synthesis, biomedical areas, materials sciences, polymers and bio-organic chemistry (Figure 2) [23,24,25,26,27,28,29,30].

The direct Diels-Alder reaction of FF or HMF with common alkenes is thermodynamically unfavorable [31,32,33], but this type of cycloaddition can be performed after decreasing the HOMO–LUMO gap through reduction of the aldehyde group into more donor functionality. Another approach is redox-neutral chemical activation through modification of aldehyde into acetal or hydrazone with the possibility of aldehyde deprotection. In general, the nature of the substituent at the C2 position in the furan ring strongly affects reactivity in DA cycloadditions; furans with electron-donating groups are well-suited as substrates, while electron-poor furans display low reactivity [34,35].

**Scheme 1:**
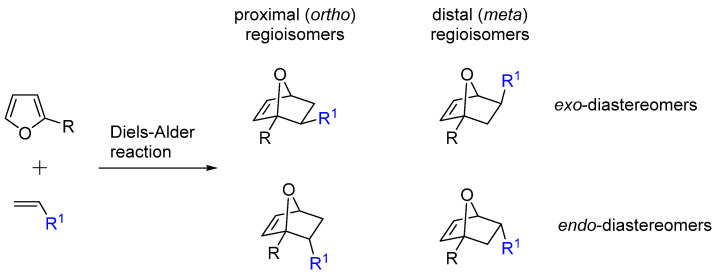
Possible regio- and diastereomers in Diels-Alder cycloaddition of C-2-substituted furans with mono-substituted alkenes.

In the case of highly active dienophiles, DA adducts may be formed under noncatalytic conditions; for other substrates, catalysis by Lewis acids is usually needed. Reactions of furans with alkene dienophiles are often characterized by facile retro-DA (rDA) reactions due to the low reactivity of furan as a diene that leads to low diastereo- and regioselectivity of the cycloaddition (Scheme 1). The orbital HOMO_diene_ and LUMO_dienophile_ energy difference seems to control the diastereomer distribution [32,36]. Charge interactions between diene and dienophile favor orthoselectivity, while steric hindrance promotes metaselectivity but without strong kinetic or thermodynamic preference for a single regioisomer [32,37].

**Scheme 2:**
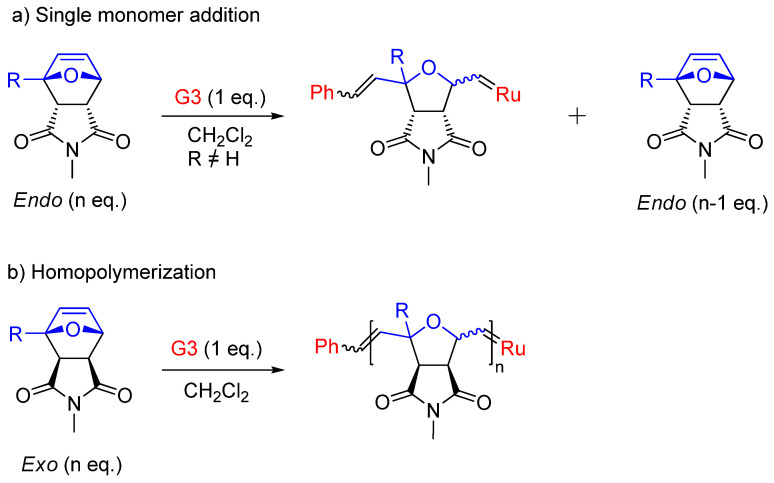
Different reactivities of *endo* (**a**) and *exo* (**b**) furan-derived oxanorbornanes in Ru-catalyzed ring-opening metathesis polymerization. R = H, Me, *n*-propyl or *n*-pentyl. G3 = third generation Grubbs catalyst.

Information about the selectivity of DA reactions is helpful to scientists in many fields, including medical and pharmaceutical research, polymer development and materials science. The regio- and diastereoselectivity of DA cycloaddition are important parameters for the high-yielding synthesis of chemically pure products, especially in the development of drugs, because diastereomers may exhibit different biological activities [38]. The *endo-* and *exo*-DA adducts have different steric properties and convert to furan and alkene components at different temperatures, which may be important in the development of various dynamic systems [39,40]. Moreover, the stereo structure of cyclic alkenes may influence the reactivity in ring-opening metathesis polymerization used for the synthesis of stereoregular polymers [41]. This difference for furan-derived oxanorbornanes was clearly demonstrated by Kilbinger and coworkers. They showed in several examples that furan/maleimide DA adducts react quickly and selectively with the G3 catalyst, resulting in the formation of monomolecular carbene complexes that display low reactivity with the second molecule of oxanorbornane (both *endo* or *exo*) due to unfavorable steric factors (Scheme 2a). In contrast, *exo*-oxanorbornane counterparts undergo efficient homopolymerization under the same reaction conditions (Scheme 2b) [41].

Several approaches may be used to increase the regio- and diastereoselectivity of DA reactions: fine-tuning of steric and electronic properties of dienes or dienophiles; variation of reaction conditions such as temperature, time, type of solvent and pressure; and catalysis by Lewis acids. Generally, for furan/alkene cycloadditions, *exo* isomers are more stable and form under thermodynamic control of the reaction (at high temperature), while *endo* isomers are kinetically preferred [36,42,43,44].

In this review, we summarized the recent literature about the regio-, stereo- and diastereoselectivity of intermolecular Diels-Alder (IMDA) cycloadditions of simple furfural derivatives with alkenes used for the synthesis of cyclic aliphatic or aromatic products. Some aspects, such as the influence of a catalyst or solvent, the type of diene and dienophile and, in some cases, comparison with other furanic substrates, were highlighted. Several reviews have covered the synthetic potential of biobased furans for the production of biofuels, chemicals and materials [10,11,15,18,30,45,46,47,48,49,50,51,52,53,54,55,56,57,58,59], as well as the mechanisms and selectivity of DA cycloadditions [60,61,62,63,64]. These discussions will not be repeated here. Instead, a dedicated survey of the literature focused on the selectivity of IMDA cycloadditions of FF derivatives with alkenes (which has not been previously reported) will be provided here.

## 2. Selectivity of Diels-Alder Cycloaddition with Furfural Derivatives as Substrates

### 2.1. 2-Methylfuran

2-Methylfuran (2-MF) is the simplest 2-substituted furan produced by the reduction of the aldehyde group in FF. The selectivity of IMDA reactions of 2-MF with common cyclic and acyclic alkenes is presented in Table 1 and Table 2. Noncatalytic reactions of 2-MF with maleic or citraconic anhydride led to cycloadducts with *exo* configurations even at room temperature (Table 1, entries 1–3). The current literature provides scarce information about the selectivity of reactions of 2-MF with maleimides under kinetic conditions. In the case of maleimides reacting with 2-MF at room temperature, the formation of >20% *endo* isomer was observed (entry 4), while at temperatures more than 60 °C, exclusive formation of the *exo* isomer was found for most maleimides (Table 1). However, in a water medium for some *N*-substituted maleimides, the content of *endo* isomers was higher even under high temperature (entries 8, 10). For *N*-carboxyethyl maleimide reacting with furan, 2-MF or 2,5-dimethylfuran, the best exoselectivity was obtained in the case of furan, while 2,5-dimethylfuran showed the best endoselectivity under kinetic conditions (entries 16–19) [65]. The cycloadduct of 2-MF with *N*-phenyl maleimide was isolated in a pure, optically active form with 90% *ee* using dynamic enantioselective crystallization by continuous suspension in heptane or hexane solution with glass beads at 80 °C in the presence of trifluoroacetic acid (TFA) to accelerate the deracemization (entry 13) [44].

**Scheme 3:**
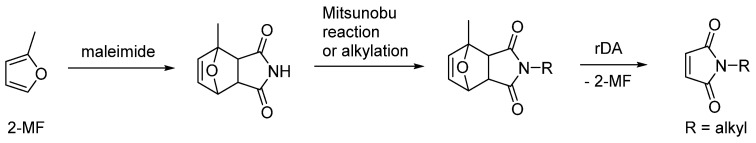
Synthesis of *N*-substituted maleimides from 2-MF and maleimide using the DA approach.

An important possible application of 2-MF is the protection of double bonds in functionalized alkenes against nucleophiles using the DA reaction. For example, modification of the 2-MF/maleimide DA adduct by alkylation or a Mitsunobu reaction, followed by thermal deprotection, was used for the synthesis of *N*-alkylated maleimides (Scheme 3) [69,70].

Representative reactions of 2-MF with acyclic alkenes containing one or two electron-withdrawing groups (EWGs) are covered in Table 2. High endoselectivity was obtained for the HfCl_4_-catalyzed reaction of 2-MF with dimethyl maleate at low temperatures (Table 2, entries 1, 2). However, under the same conditions, benzyl acrylate showed exoselectivity for cycloaddition (entries 7, 8). An adduct of 2-MF and trans-4,4,4-trifluorocrotonic acid formed with high regio- and diastereoselectivity (entry 3). An enantioselective version of DA reactions with some fluorinated alkene dienophiles was implemented using chiral oxazaborolidine organocatalysts, which affords corresponding chiral oxabicyclic products with high yields and selectivity (entries 4–6). In the case of acrylonitrile reacting with 2-MF, regio- and diastereoselectivity was poor even in the presence of Lewis acid catalysts (entries 9, 10). Orthoadducts of 2-MF with 1-cyanovinyl acetate or 2-chloroacrylonitrile that are favored over *meta*-isomers due to electronic reasons were obtained under kinetic conditions with high regioselectivity (entries 11–15). A shift towards *endo*-products was found for reactions of 2-MF with allenic esters in the presence of Eu(fod) as the catalyst (entries 16–19).

**Table 2:**
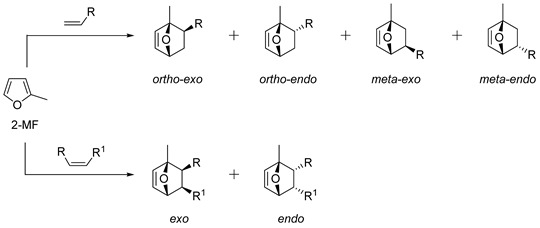
IMDA cycloadditions of 2-MF with acyclic alkenes.

№	Dienophile	Conditions	Selectivity	Yield of Adducts (%), [Ref.]
1	Dimethyl maleate	HfCl_4_, CH_2_Cl_2_, −30 °C	*Endo*/*exo* 84:16	94, [76]
2	Dimethyl maleate	HfCl_4_, CH_2_Cl_2_, −50 °C	*Endo*/*exo* > 98:2	82, [76]
3	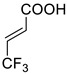	22 °C	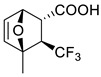	90, [77]
4	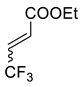	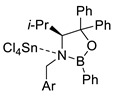 Ar = 1-naphthyl (cat.), CH_2_Cl_2_, −78 °C	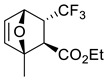 99 *de*, 99 *ee*	99, [78]
5	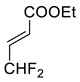	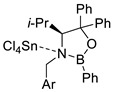 Ar = 1-naphthyl (cat.), CH_2_Cl_2_, −78 °C	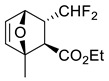 99 *de*, 99 *ee*	74, [78]
6	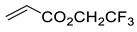	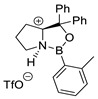 (cat.), CH_2_Cl_2_, −78 °C	*Ortho (endo*/*exo* 94:6), 98 *ee* (for *endo* isomer)	74, [79]
7	Benzyl acrylate	HfCl_4_, CH_2_Cl_2_, −30 °C	*Endo*/*exo* 28:72 (mixture of regio isomers)	84, [76]
8	Benzyl acrylate	HfCl_4_, CH_2_Cl_2_, −50 °C	*Endo*/*exo* (31:69) (mixture of regio isomers)	85, [76]
9	Acrylonitrile	ZnI_2_, neat, 50 °C	N.d.	69, [80]
10	Acrylonitrile	Neat, 60 °C	*Ortho* 66 (*endo*/*exo* 61:39), *meta* 34 (*endo*/*exo* 56:44)	69, [31,32]
11	1-Cyanovinyl acetate	ZnI_2_, neat, 0 °C, 8 days	*Ortho* (*endo/exo* 1:1) ^2^	52, [81]
12	1-Cyanovinyl acetate	ZnI_2_, neat, 20 °C, 26 h	*Ortho endo* ^2^	17, [81]
13	1-Cyanovinyl acetate	ZnI_2_, neat, RT, 24 h	*Ortho* (*endo*/*exo* 3:1) ^2^	30, [82]
14	1-Cyanovinyl acetate	MgI_2_, neat, RT, 24 h	*Ortho* (*endo*/*exo* 4:1) ^2^	57, [82]
15	2-Chloroacrylonitrile	ZnI_2_, neat, 0 °C	*Ortho/meta* 10:1 (mixture of *endo*/*exo*)	91 ^1^, [83]
16	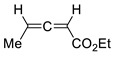	Benzene, reflux	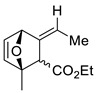 *Endo*/*exo* 1,1:1	70, [84]
17	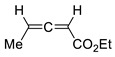	Eu(fod), RT	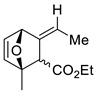 *Endo*/*exo* 2,1:1	80, [84]
18	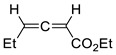	Eu(fod), RT	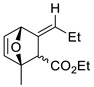 *Endo*/*exo* ~2,8:1	80, [84]
19	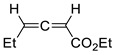	Benzene, reflux	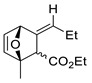 *Endo*/*exo* ~1:1	73, [84]
20	Itaconic anhydride 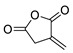	Neat, 23 °C	*Ortho* (*endo*:*exo*)/*meta* (*endo*:*exo*) 3:1/11:8 ^3^	13 ^4^, [85]

^1^ Yield of DA adduct after hydrogenation. ^2^ *Endo*- and exoconformation with regard to the position of the OAc group. ^3^ Structure of regio- and diastereomers in DA cycloaddition of C-2-substituted furans with itaconic anhydride are provided in Scheme 5. ^4^ Was detected by NMR. N.d.—not determined.

### 2.2. Furanic Acetals

With rare exceptions, furfural does not react with dienophiles, but the introduction of aldehyde groups by DA reaction may be performed using an acetalization strategy that reduces the electron-withdrawing character of the carbonyl group. Table 3 highlights the results of reactions of furanic acetals with cyclic and linear alkenes. Literature data about the stereoselectivity of reactions of furanic acetals with cyclic alkenes are scarce. Predominant formation of endoadducts under kinetic conditions was detected by NMR when *N*-methyl maleimide was used as a dienophile (entry 1). For reactions of furfural acetals with mono-substituted acyclic alkenes, regioselectivity significantly depended on the type of substrates and reaction conditions. For dioxolane acetal reacting with methyl vinyl ketone, methyl acrylate or acrolein at 60 °C, a mixture of regio- and stereoisomers was obtained with predominant *meta*- and endoselectivity. In the case of acrylonitrile reacting with furanic acetals, the selectivity of cycloadditions was poor even in the presence of Lewis acid catalysts (entries 5–9). For the ZnCl_2_-catalyzed reaction of ethylthioacetal with acrylonitrile at 30 °C, 91% orthoselectivity and moderate endoselectivity were observed (entry 10). According to DFT calculations, the regioselectivity of reactions of furanic acetals with alkenes is a result of two opposite factors: charge interactions between the furan and alkene favor orthoselectivity, while steric factors promote metaselectivity [32].

### 2.3. Functionalized Furfural Derivatives

Mild reduction of the aldehyde group in FF is a path to important furanic building blocks furfuryl alcohol (FA) and furfuryl amine (FAM), which are widely used for the development of functional or dynamic molecular and biomolecular systems. Examples of possible areas of applications include but are not limited to the synthesis of biologically active compounds [87,88,89,90], oxanorbornane-based amphiphiles [91,92,93,94], supramolecular systems [95], self-assemblies [96], self-healing polymers and other dynamic systems [28].

The diastereoselectivity of DA reactions of FA, FAM and some common derivatives with cyclic and acyclic alkenes is shown in Table 4, Table 5 and Table 6. Preferable formation of exoadducts was observed for reactions of maleic and citraconic anhydrides with selected furanic substrates even at low temperatures (Table 5 and Table 6), except for the vinylated derivative of FA, which showed preferable endoselectivity (Table 5, entries 5–10).

**Scheme 4:**
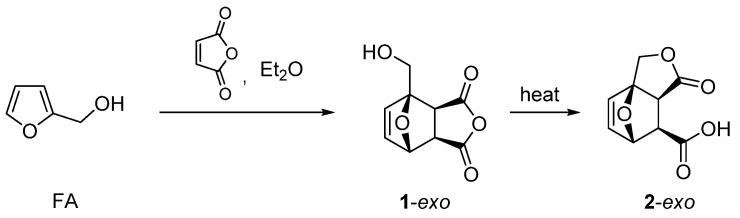
Formation of lactone **2**-*exo* after DA reaction of FA with maleic anhydride.

The adduct of FA with maleic anhydride (**1**-*exo*) is unstable and undergoes irreversible intramolecular cyclization during storage or warming, yielding the corresponding thermodynamically stable lactone **2**-*exo* (Scheme 4) [102].

The diastereoselectivity of the reactions with *N*-alkyl- and *N*-benzyl-substituted maleimides was in accordance with typical kinetic profiles demonstrating a shift towards *endo-* and *exo*-products under kinetic or thermodynamic conditions, respectively (Table 4, Table 5 and Table 6). However, this relationship was disrupted for some *N*-aryl maleimides reacting with various furanic substrates under both kinetic and thermodynamic conditions. For example, the diastereoselectivity of the cycloaddition of vinyl-substituted FA and *N*-Ph-maleimide shifted from a 1:2.8 *endo*/*exo* ratio under kinetic conditions to Et_2_O to a 4:1 *endo*/*exo* ratio in toluene at 80 °C (Table 5, entries 11, 12).

**Table 5:**
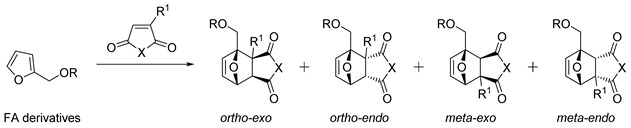
IMDA cycloadditions of FA derivatives with cyclic alkenes.

№	R	Dienophile	Conditions	Selectivity	Yield of Adducts (%), [Ref.]
1	Allyl	*N*-Me-maleimide	Toluene, 50 °C, 24 h	N.d.	65 (*endo*), [103]
2	Allyl	*N*-Ph-maleimide	Toluene, 50 °C, 24 h	N.d.	26 (*exo*), [103]
3	Bn	Maleic anhydride	Toluene, RT, 3 days	*Exo*	43, [91]
4	Bn	Citraconic anhydride 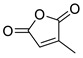	15 kbar, CH_2_Cl_2_, 60 h	*Exo* (*ortho*/*meta* 5:7)	31 ^1^, [68]
5	Vinyl	Maleic anhydride	Et_2_O, 22‒24 °C, 48 h	*Endo*	72, [104]
6	Vinyl	Maleic anhydride	Et_2_O, 35 °C, 48 h	*Endo*/*exo* 8:1	66, [104]
7	Vinyl	Maleic anhydride	THF, 22‒24 °C, 90 h	*Endo*/*exo* 8:1	66, [104]
8	Vinyl	Maleic anhydride	MeCN, 22‒24 °C, 48 h	*Endo*/*exo* 4:1	68, [104]
9	Vinyl	Maleic anhydride	Toluene, 22‒24 °C	*Endo*/*exo* 12:1	64, [104]
10	Vinyl	Maleic anhydride	Toluene, 80 °C	*Endo*/*exo* 4:1	66, [104]
11	Vinyl	N-Ph-maleimide	Et_2_O, 22‒24 °C	*Endo*/*exo* 1:2.8	47, [104]
12	Vinyl	N-Ph-maleimide	Toluene, 80 °C	*Endo*/*exo* 4:1	66, [104]
13	Ac	Maleic anhydride	Et_2_O, 25 °C, 7 days	*Exo*	34, [105]
14	Ac	Maleic anhydride	Toluene, RT, 97 h	*Exo*	74, [88]
15	Ac	Citraconic anhydride 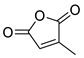	15 kbar, CH_2_Cl_2_, 60 h	*Exo* (*ortho*/*meta* 6:5)	59 ^1^, [68]
16	Ac	*N*-Me-maleimide	CH_2_Cl_2_, 23 °C	*Endo*/*exo* 77:23	N.d., [86]
17	Ac	*N*-Dodecylmaleimide	THF, 23 °C	*Endo*/*exo* 64:36	N.d., [86]
18	Ac	*N*-Ph-maleimide	CH_2_Cl_2_, 23 °C	*Endo*/*exo* 65:35	N.d., [86]
19	Ac	*N*-(*p*-Nitrophenyl)maleimide	CH_2_Cl_2_, 23 °C	*Endo*/*exo* 55:45	N.d., [86]
20	Ac	*N*-(*p*-Methoxyphenyl)maleimide	CH_2_Cl_2_, 23 °C	*Endo*/*exo* 67:33	N.d., [86]
21	Ac	*N*-(Methoxy-2-propyl)maleimide	CH_2_Cl_2_, 23 °C	*Endo*/*exo* 76:24	N.d., [86]
22	Ac	*N*-(2-Methoxyethyl)maleimide	CH_2_Cl_2_, 23 °C	*Endo*/*exo* 75:25	N.d., [86]
23	Bz	Maleic anhydride	Toluene, 80 °C, 456 h	*Exo*	46, [88]
24 ^2^	Bz	Maleic anhydride	Et_2_O, 24 °C, 24 h	*Endo*	84, [106]
25	Bz	*N*-Me-maleimide	CH_2_Cl_2_, 23 °C	*Endo*/*exo* 70:30	N.d., [86]
26	Bz	*N*-Dodecylmaleimide	THF, 23 °C	*Endo*/*exo* 63:37	N.d., [86]
27	CO*^i^*Bu	*N*-Pr-maleimide	CHCl_3_, 55 °C	*Endo*/*exo* 60:40	N.d., [107]
28	CO*^i^*Bu	*N*-*^i^*Bu-maleimide	CHCl_3_, 55 °C	*Endo*/*exo* 45:55	N.d., [107]
29	CO*^i^*Bu	*N*-*^t^*Bu-maleimide	CHCl_3_, 55 °C	*Endo*/*exo* 51:49	N.d., [107]
30	CO*^i^*Bu	*N*-Bn-maleimide	CHCl_3_, 55 °C	*Endo*/*exo* 44:56	N.d., [107]
31	CO*^i^*Bu	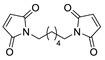	CHCl_3_, 55 °C	*Endo*/*exo* 26:74	N.d., [107]
32	CO*^i^*Bu	*N*-(*2*-Methylphenyl)-maleimide	CHCl_3_, 55 °C	*Endo*/*exo* 67:33	N.d., [107]
33	CO*^i^*Bu	BMI	CHCl_3_, 55 °C	*Endo*/*exo* 19:81	N.d., [107]
34	CO*^t^*Bu	*N*-Me-maleimide	CH_2_Cl_2_, 23 °C	*Endo*/*exo* 71:29	N.d., [86]
35	CO*^t^*Bu	*N*-Dodecylmaleimide	THF, 23 °C	*Endo*/*exo* 62:38	N.d., [86]

^1^ Yield of DA adduct after hydrogenation. ^2^ BHMF dibenzoate as a substrate. N.d.—not determined.

**Scheme 5:**
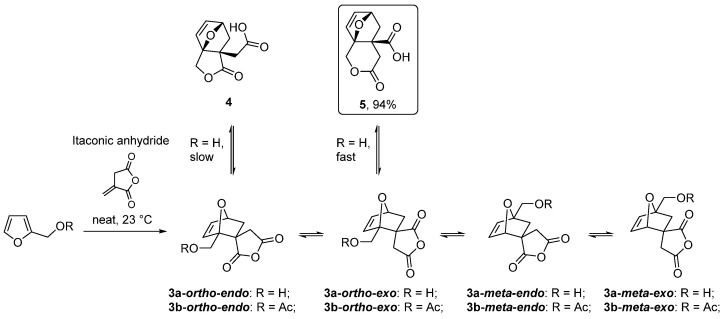
Diels—Alder reactions of FA and FA acetate with itaconic anhydride.

Information about the regio- and diastereoselectivity of functional FF derivatives with acyclic alkenes is scarce. A mixture of regio- and diastereoisomers with approximately equal distribution was detected after the noncatalytic reaction of FA with acrylonitrile (Table 4, entry 14). A mixture of regio- and diastereomers with *ortho* (*endo*:*exo*)/*meta* (*endo*:*exo*) 2:1/8:6 ratio was formed from itaconic anhydride reacting with FA acetate (Scheme 5) [85]. However, unfavorable thermodynamic parameters of cycloaddition with this dienophile were overcome using FA as a substrate, where proximal (*ortho*) DA adducts undergo further intramolecular cyclization, shifting the reaction equilibrium towards metastable lactone **5,** which was isolated in 94% yield (Scheme 5) [85].

Overall, the diastereoselectivity of DA reactions of alkenes with FF derivatives containing donor substituents at the C2 position is not always predictable, because it strongly depends on the structure of both the diene and dienophile. More predictable diastereoselective construction of functionalized oxabicyclic structures may be performed using HMF-derived 2,5-disubstituted furans that predominantly react with cyclic alkenes with high endoselectivity (Table 4, entries 1–2; Table 5, entry 24) [33,43,106,108].

**Table 6:**
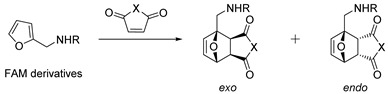
IMDA cycloadditions of FAM derivatives with cyclic alkenes.

№	R	Dienophile	Conditions	Selectivity	Yield of Adducts (%), [Ref.]
1	Ac	Maleic anhydride	Et_2_0, 23 °C	*Exo*	100, [109]
2	Ac	Maleimide	H_3_BO_3_/PEG-400, 90 °C	*Exo*	84, [110]
3	Ac	*N*-Ph-maleimide	H_3_BO_3_/PEG-400, 90 °C	*Exo*	78, [110]
4	Ac	*N*-(4-Chlorobenzyl)maleimide	H_3_BO_3_/PEG-400, 90 °C	*Exo*	92, [110]
5	Boc ^1^	Maleic anhydride	Toluene, 50 °C	*Exo*	94, [111]
6	Boc ^1^	Thiomaleic anhydride	Benzene, RT	*Exo*	68, [112]
7	Boc ^1^	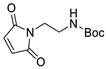	EtOAc, reflux	*Endo*/*exo* (1:3.4)	85, [113]

^1^ *tert*-Butyloxycarbonyl.

Examples of DA reactions of furfural derivatives containing acceptor-type substituents with alkenes are rare. After the reaction of 2-furoic acid with β-alanine-substituted maleimide, only a small amount of one isomer was detected at 40 °C after 128 h [26]. Interestingly, a very low equilibrium constant for this reaction was observed in DMF media, while the equilibrium constant in water was at least two orders of magnitude greater. This difference was explained by the statement that water has a significant effect on the entropy of the reaction. The model reaction of methyl furoate with 1,6-bis(*N*-maleimido)hexane was investigated by NMR. Only approximately 20% conversion was detected after 4 days at 70 °C in a DMSO-*d*_6_ medium [35]. However, despite the low reactivity of furans with acceptor substituents, dynamic materials containing furanic ester-[35] or oxime-[114] functionalized polymers and maleimide functionalities showed moderate self-healing efficiency based on the DA reaction.

Bruijnincx and coworkers reported a new strategy for the direct introduction of furans containing aldehyde groups into DA cycloaddition [34]. Reactions of furanic aldehydes with water-soluble maleimides at 60 °C in a water medium led to the formation of DA adducts with good selectivity (Table 7). In the case of furfural, good exoselectivity of cycloaddition was achieved, while for some HMF derivatives, endoselectivity was preferable. In-water formation of the DA adduct was also detected for 2-acetylfuran, which reacts with *N*-methylmaleimide with the formation of only the exoadduct (entry 9). DFT calculations showed that the formation of furan/maleimide DA adducts through hydration of the aldehyde group is thermodynamically possible if hydration occurs both prior to (which increases the rate of the forward DA reaction) or after the cyclization step (which decreases the rate of the retro-DA reaction) [34].

## 3. Regioselectivity in the Synthesis of Aromatics Using the IMDA Reaction of Furfural Derivatives with Alkenes

**Scheme 6:**
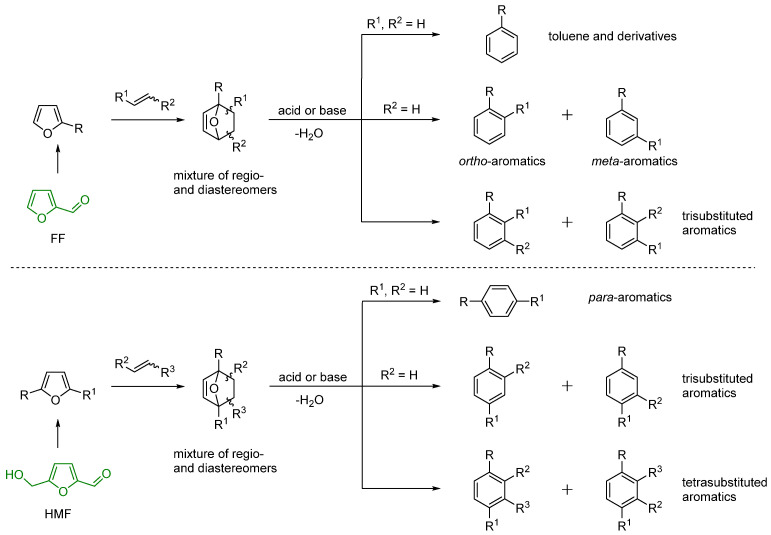
Aromatization of furan/alkene DA adducts as a route towards biobased aromatics.

The dehydration of furan/alkene adducts is an important sustainable approach to accessing renewable aromatic chemicals (Scheme 6) [7,30,37,115,116,117]. Utilization of HMF-derived C6 renewable furans (especially 2,5-dimethylfuran or 2,5-furandicarboxylic acid) provides access to *para*-substituted aromatics (as a route towards “green” polymers) and various polysubstituted aromatic products (Scheme 6) [116]. The presence of only one substituent in furfural increases the diversity of possible aromatic products to *ortho*- and *meta*-xylylene derivatives as well as various 1,2,3-trisubstituted compounds (Scheme 6).

Several approaches were used for the construction of aromatic rings using furan/alkene DA reactions starting from furanic, oxanorbornene or oxanorbornane furfural-derived compounds. For some furanic and alkene substrates, dehydration occurs spontaneously following the DA reaction stage. The tandem Diels-Alder cycloaddition/dehydration reaction of 2-MF with ethylene is an important approach to renewable toluene (Table 8). This type of DA cycloaddition is thermodynamically difficult and therefore requires the use of a catalyst, high temperature and pressure. Heterogeneous Brønsted-acidic catalysts, mainly zeolites or MOFs, are beneficial for these reactions [118]. Significant problems include side reactions such as the formation of furanic dimers (benzofurans), larger oligomers, products of furan hydrolysis and other reactions [115,118,119,120]. The introduction of acrylic acid instead of ethylene in reactions with 2-MF over zeolites or using ionic liquid catalysts showed good efficiency in the formation of aromatics [121]. Fast pyrolysis of a mixture of 2-MF and propylene using various zeolites under continuous flow conditions gives a mixture of monocyclic and polycyclic aromatic hydrocarbons with low selectivity [122].

Furfural dimethyl hydrazone reacts with active dienophiles such as maleic anhydride or maleimides, yielding corresponding arene derivatives through noncatalytic in situ DA cycloaddition followed by spontaneous dehydration (Table 9) [126,127,128]. One-pot synthesis of arenes starting from furfural using a hydrazine strategy was carried out with good yields in water (entries 7–11) [129].

**Scheme 7:**
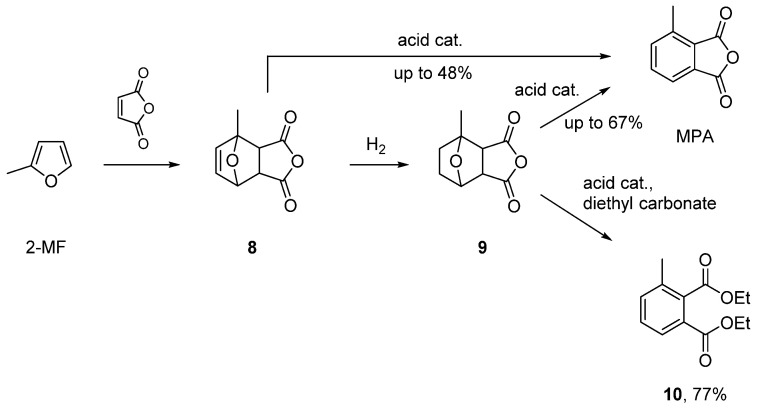
Synthesis of arenes by aromatization of 2-MF-derived tricycles.

Acid-catalyzed dehydration of furan-derived oxanorbornenes to aromatic products requires strong reaction conditions and therefore may be used only for a narrow range of substrates. Renewable 3-methylphthalic anhydride (MPA) was obtained using acid-catalyzed dehydration of the corresponding 2-MF-derived DA adduct **8** with only 48% maximum yield (Scheme 7) [130]. An important problem in this synthetic approach is the facile retro-DA reaction, which is forced to carry out these transformations at industrially non-practical temperatures (−30 °C and lower) [124,125]. A novel approach to MPA synthesis that overcomes the problem of the rDA reaction is the introduction of oxanorbornane **9** (which is unable to recycle) instead of **8** into the aromatization stage (Scheme 7) [67,131,132]. Aromatization of **9** by solid acid catalysts led to MPA with 67% maximum yield. Some important byproducts, such as 2-methyl benzoic acid and 3-methyl benzoic acid, were also formed during this reaction, and their ratio depended on the catalyst used [67,131]. Higher selectivity of aromatization was achieved by oxidative dehydrogenation of **9** into phthalate **10** using a silicomolybdic acid catalyst in diethyl carbonate (Scheme 7) [132].

The deprotonation of DA adducts formed from 2-(furan-2-yl)-1,3-dioxolane and acrylonitrile by CH_3_ONa/DMSO superbase affords aromatic products at 30 °C with high total yield and a good *ortho*/*meta* ratio (Table 10, entries 1, 2) [31]. The study of kinetic features of the aromatization stage showed that the *meta*-adduct is more reactive than the *ortho*-isomer, which made it possible to isolate pure *meta*-adducts from the reaction mixture at 50% conversion, with subsequent regeneration of the *ortho*-isomer. Aromatization of DA adducts by *^t^*BuONa/DMSO superbase was also efficient for 2-MF and methyl group-protected FA but showed a low yield of aromatics in the case of unprotected FA (Table 10, entries 3–5) [31].

**Scheme 8:**
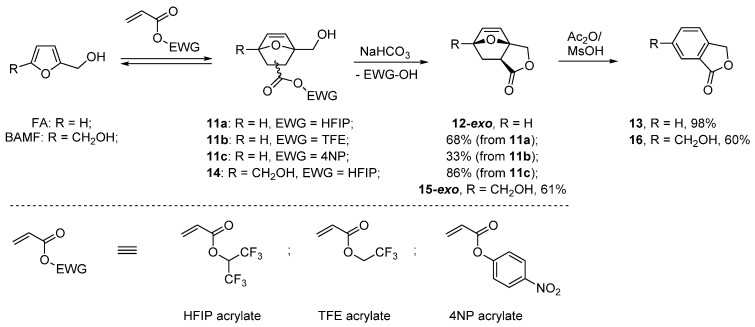
Synthesis of phthalides from furanic alcohols using a dynamic kinetic trapping strategy. HFIP = 1,1,1,3,3,3-hexafluoroisopropyl. TFE = 2,2,2-trifluoroethyl. 4NP = 4-nitrophenyl.

Recently, a new dynamic kinetic trapping strategy was developed for the construction of “drop-in” phthalide systems using tandem IMDA/lactonization and then aromatization reactions (Scheme 8) [37]. The first stage of this process is the reversible formation of unstable adducts (mixture of regio- and stereoisomers) of FA (**11a–c**) or BAMF (**14**) with acrylates substituted by EWGs (HFIP, TFE or 4NP) at an oxygen atom. The role of EWG in the dienophile was the activation of both double bonds for the IMDA reaction and the carbonyl group towards diastereoselective intramolecular cyclization and into a more thermodynamically stable *exo*-lactone (the next step). The last aromatization stage was performed using an Ac_2_O/strong acid mixture yielding phthalides **13** or **16** with maximum 98% and 60% yields, respectively.

## 4. Conclusions

The IMDA reactions of biobased furans with alkene dienophiles are an important strategy for accessing practically important products, such as fundamental building blocks, fine chemicals, biologically active compounds or various organic and hybrid dynamic systems. Based on the literature highlighted in this review, we can assume that the problem of low regio- and stereoselectivity, which significantly reduces the synthetic potential of furan/alkene DA cycloaddition in fine organic synthesis and materials development, is still not solved for many functional furfural derivatives and alkene substrates. The reactivity of furfural-derived acceptor furans towards common alkenes, as well as the synthesis and aromatization of DA adducts of functional furfural derivatives with acyclic alkenes, are very poorly represented in the current literature. However, these types of reactions are important sustainable approaches towards functional aliphatic or aromatic products and therefore require further scientific investigations.

Rapid progress in this area can be anticipated, taking into account emerging trends in sustainable development towards the incorporation of bioderived chemicals and materials into the chemical industry. The focus of this review clearly shows that selectivity issues are far from solved and do not match current requirements. More studies are needed to develop practical and easy-to-use procedures to achieve high selectivity in reactions involving simple bioderived furanic starting materials.

## Figures and Tables

**Figure 1:**
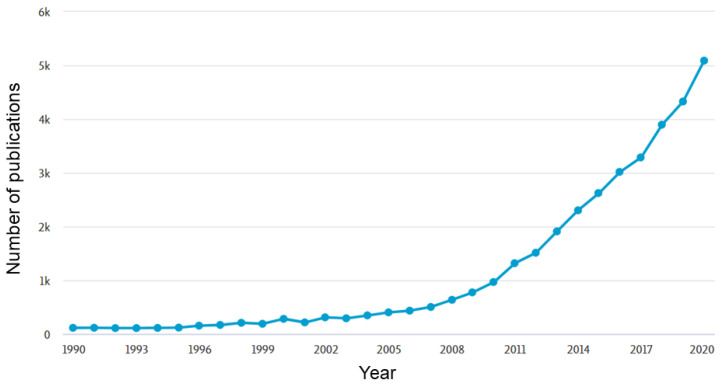
Number of publications mentioning biobased furans per year. Source: Scopus. Keyword: “furfural”.

**Table 1:**
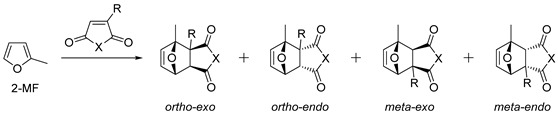
IMDA cycloadditions of 2-MF with cyclic alkenes.

№	Dienophile 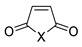	Conditions	Selectivity	Yield of DA Adducts (%), [Ref.]
1	X = O	Neat, RT, N_2_, 24 h	*Exo*	91, [66]
2	X = O	Neat, RT, 10–15 °C, 2–3 h	*Exo*	96 (crude), [67]
3	Citraconic anhydride 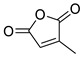	CH_2_Cl_2,_ RT, 15 kbar	*Exo* (*ortho*/*meta* 1:1)	65 ^1^, [68]
4	X = NH	Et_2_O, RT, 3 days	*Endo*/*exo* ^2^	21 (for *endo*), [69]
5	X = NH	THF, reflux, 4 h	*Exo*	94, [70]
6	X = NMe	Toluene, 90 °C	*Exo*	92, [71]
7	X = NMe	Et_2_O, 90 °C	*Exo*	66, [72]
8	X = NEt	H_2_O, 65 °C	*Endo*/*exo* 1.4:1	100, [73]
9	X = N(*^t^*Bu)	H_2_O, 65 °C	*Exo*	100, [73]
10	X = NPh	H_2_O, 65 °C	*Endo*/*exo* 1.6:1	100, [73]
11	X = NPh	4:1 Toluene/benzene, RT, 1.1 GPa	*Endo*/*exo* 1.66:1	85, [74]
12	X = NPh	CDCl_3_, 60 °C	*Exo* with traces of *endo*	90, [44]
13	X = NPh	Hexane or heptane, TFA, glass beads, 80 °C, 5–8 days ^3^	(-)-*Exo*, 86–90 *ee*	80, [44]
14	X = NPhF_5_	Neat, reflux	*Exo*	50, [75]
15	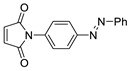	THF, 65 °C	*Exo*	64, [70]
16	X = NCH_2_CH_2_COOH	CHCl_3_, 38 °C, 5 days	*Endo*/*exo* 28:72	100, [65]
17 ^4^	X = NCH_2_CH_2_COOH	CHCl_3_, 38 °C, 5 days	*Exo*	100, [65]
18 ^5^	X = NCH_2_CH_2_COOH	CH_2_Cl_2_, RT, overnight	*Endo*/*exo* 78:22	100, [65]
19 ^5^	X = NCH_2_CH_2_COOH	CH_3_CN, 60 °C, 6 h	*Endo*/*exo* 22:78	100, [65]

^1^ Yield of DA adduct after hydrogenation. ^2^ Ratio of diastereomers was not provided. ^3^ The reaction was conducted under dynamic enantiomeric crystallization conditions. ^4^ Furan as a substrate. ^5^ 2,5-Dimethylfuran as a substrate.

**Table 3:**
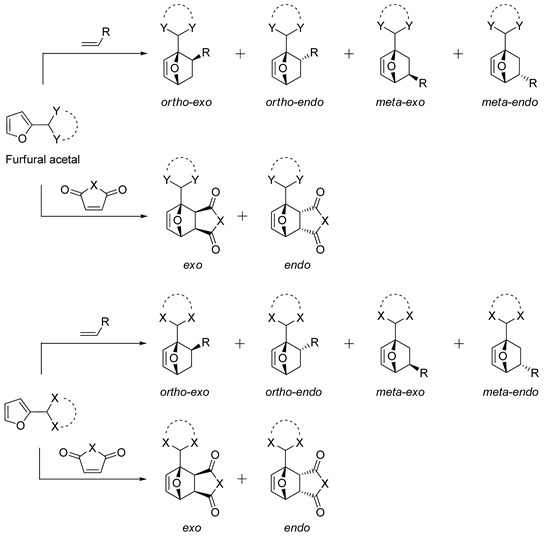
IMDA cycloadditions of furfural acetals with alkenes.

№	Furfural Acetal	Dienophile	Conditions	Selectivity	Yield of Adducts (%), [Ref.]
1	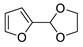	*N*-Methylmaleimide	CH_2_Cl_2_, 23 °C	*Endo*/*exo* 87:13	N.d., [86]
2	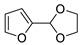	Methyl vinyl ketone	Neat, 60 °C	*Ortho* 13 (*endo*/*exo* 74:26), *meta* 87 (*endo*/*exo* 65:35)	36, [32]
3	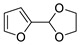	Methyl acrylate	Neat, 60 °C	*Ortho* 33 (*endo*/*exo* 87:13), *meta* 67 (*endo*/*exo* 77:23)	40, [32]
4	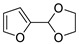	Acrolein	Neat, 60 °C	*Ortho* 38 (*endo*/*exo* 71:29), *meta* 62 (*endo*/*exo* 43:57)	28, [32]
5	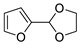	Acrylonitrile	Neat, 60 °C, 120 h	*Ortho* 48 (*endo*/*exo* 72:28), *meta* 52 (*endo*/*exo* 42:58)	76, [32]
6	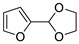	Acrylonitrile	ZnCl_2_, neat, 60 °C	*Ortho* 50 (*endo*/*exo* 70:30), *meta* 50 (*endo*/*exo* 56:44)	75, [32]
7	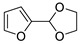	Acrylonitrile	ZnI_2_, neat, 60 °C	*Ortho* 53 (*endo*/*exo* 70:30), *meta* 67 (*endo*/*exo* 60:40)	75, [31]
8	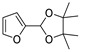	Acrylonitrile	ZnCl_2_, neat, 60 °C	*Ortho* 43 (*endo*/*exo* 85:15), *meta* 57 (*endo*/*exo* 56:44)	68, [32]
9	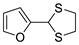	Acrylonitrile	ZnCl_2_, neat, 60 °C	*Ortho* 39 (*endo*/*exo* 67:33), *meta* 61 (*endo*/*exo* 54:46)	67, [32]
10	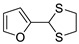	Acrylonitrile	ZnCl_2_, neat, 30 °C	*Ortho* 91 (*endo*/*exo* 66:33), *meta* 9 (*endo*/*exo* 53:47)	73, [32]
11	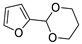	Acrylonitrile	ZnCl_2_, neat, 60 °C	*Ortho* 53 (*endo*/*exo* 60:40), *meta* 47 (*endo*/*exo* 54:46)	81, [32]
12	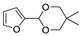	Acrylonitrile	ZnCl_2_, neat, 60 °C	*Ortho* 52 (*endo*/*exo* 62:38), *meta* 48 (*endo*/*exo* 56:44)	85, [32]

N.d.—not determined.

**Table 4:**
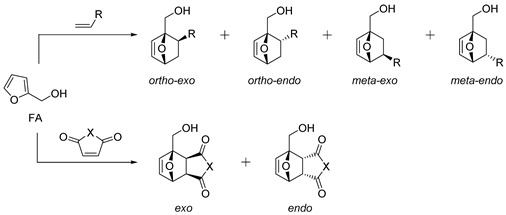
IMDA cycloadditions of FA with alkenes.

№	Dienophile	Conditions	Selectivity	Yield of Adducts (%), [Ref.]
1 ^1^	Maleimide	Ethyl acetate, 24 °C	*Endo*/*exo* 96:4	87, [33]
2 ^2^	Maleimide	Ethyl acetate, 24 °C	*Endo*/*exo* 97:3	42, [33]
3	*N*-Me-maleimide	Et_2_O, 90 °C	*Endo*/*exo* 21:79	43, [72]
4	*N*-Bn-maleimide	CH_3_CN, 35 °C	*Endo*/*exo* 70:30	75, [96]
5	*N*-Propargylmaleimide	CH_3_CN, 35 °C	*Endo*/*exo* 80:20	72, [96]
6	*N*-(2-Hydroxymethyl)maleimide	Ethyl acetate, 80 °C	*Exo*	76, [97]
7	*N*-(2-Hydroxyethyl)maleimide	Benzene, reflux	*Exo*	86, [98]
8	*N*-(3-Hydroxypropyl)maleimide	Toluene, 80 °C	*Endo*/*exo* 30:70 ^3^	77, [99]
9	*N*-(4-Hydroxyphenyl)maleimide	Acetone, 55 °C	*Exo*	71, [40]
10	*N*-(4-Hydroxyphenyl)maleimide	Acetonitrile, 35 °C	*Endo*/*exo* 80:20	N.d., [40]
11	*N*-(*p*-Methoxyphenyl)maleimide	CH_3_CN, 40 °C, 18 h	Mostly *exo*	89, [100]
12	*N*-(*p*-Nitrophenyl)maleimide	CH_3_CN, 60 °C	*Endo*/*exo* 70:23	52, [100]
13	BMI ^4^	Toluene, 75–80 °C, 2 days	Mostly *exo*	92, [101]
14	Acrylonitrile	Neat, 60 °C	*Ortho* 56 (*endo*/*exo* 69:31), *meta* 44 (*endo*/*exo* 56:44)	81, [32]
15	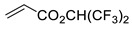	Neat, RT, 96 h	N.d.	66, [37]

^1^ 2,5-bis(Hydroxymethyl)furan (BHMF) as a substrate. ^2^ 2,5-bis(Acetoxymethyl)furan (BAMF) as a substrate. ^3^ Slowly transformed to the *exo* isomer over a period of several months. ^4^ 4,4′-bis(Maleimido)diphenylmethane. N.d.—not determined.

**Table 7:**
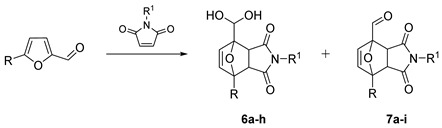
Direct DA reaction of furanic aldehydes with maleimides in water medium.

№	Furanic Substrate	R^1^	Products, Selectivity ^1^
1	R = H	Me	**6a** (*endo*/*exo* 18:40), **7a** (*endo*/*exo* 1:3)
2	R = H	H	**6b** (*endo*/*exo* 8:30), **7b** (*endo*/*exo* 0:0)
3	R = H	Et	**6c** (*endo*/*exo* 8:28, **7c** (*endo*/*exo* 1:6)
4	R = H	*^n^*Pr	**6d** (*endo*/*exo* 1:7), **7d** (*endo*/*exo* 1:11)
5	R = H	Ph	**6e** (*endo*/*exo* 0:1), **7e** (*endo*/*exo* 1:5)
6	R = Me	Me	**6f** (*endo*/*exo* 3:8), **7f** (*endo*/*exo* 0:3)
7	R = CH_2_OH	Me	**6g** (*endo*/*exo* 37:13), **7g** (*endo*/*exo* 0:0)
8	R = CH_2_OMe	Me	**6h** (*endo*/*exo* 7:5), **7h** (*endo*/*exo* 3:3)
9	2-Acetylfuran	Me	**7i** (*endo*/*exo* traces:32)

Reaction conditions: H_2_O, 60 °C, 16 h. ^1^ Determined by ^1^H NMR (data were obtained from reference [34]).

**Table 8:**
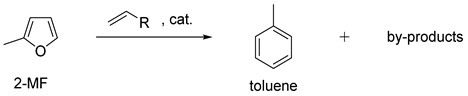
Synthesis of toluene by DA reaction of 2-MF with alkenes.

№	R	Conditions	Products Yield (%), [Ref.]
1	H	H-BEA zeolite, heptane, 62 bar, 250 °C	Toluene (46%), [119]
2	H	H-Beta-22 zeolite, 300 °C, 20 h	Toluene (50%), [123]
3	COOH	Bi-BTC, 160 °C, 24 h	Toluene (65%), 2-methyl benzoic acid (23%), [121]
4	COOH	[Emim]NTf_2_, Sc(OTf)_3_, 15 °C, 0.5 h	Toluene (12%), 2-methyl benzoic acid (2%), 3-methyl benzoic acid (9%), [124]
5	COOH	[BSO_3_HMIm]HSO_4_, 100 °C, 2h	Toluene (12%), 2-methyl benzoic acid (30%), 3-methyl benzoic acid (3%), [125]

**Table 9:**
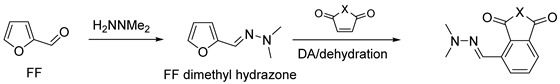
Preparation of phthalimides from furfural using a hydrazine strategy.

№	Substrates	Conditions	Yield of Aromatic Product, [Ref.]
5	2-Furaldehyde dimethylhydrazone, maleic anhydride	CHCl_3_, RT	94, [126]
6	2-Furaldehyde dimethylhydrazone, *N*-Et-maleimide	CHCl_3_, RT	90, [126]
7	2-Furaldehyde, *N,N*-dimethylhydrazine, *N*-Et-maleimide	H_2_O, 50 °C	97, [129]
8	2-Furaldehyde, *N,N*-dimethylhydrazine, maleimide	H_2_O, 50 °C	86, [129]
9	2-Furaldehyde, *N,N*-dimethylhydrazine, *N*-cyclopropylmaleimide	H_2_O, 50 °C	80, [129]
10	2-Furaldehyde, *N,N*-dimethylhydrazine, *N*-Ph-maleimide	H_2_O, 50 °C	73, [129]
11	2-Furaldehyde, *N,N*-dimethylhydrazine, *N*-(4-Methylbenzyl)maleimide	H_2_O, 50 °C	68, [129]

**Table 10:**
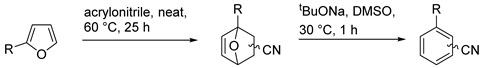
Preparation of aromatics by base-catalyzed dehydration of acrylonitrile-derived oxanorbornenes.

№	Oxanorbornene	Yield of DA Adducts ^1^	Yield of Aromatic Products ^1^
1	R = dioxolane acetal	76 (*ortho/meta* ~1:1) ^2^	84 (*ortho/meta* ~1:1.5)
2	R = dioxolane acetal	76 (*ortho/meta* ~1:1)	86 (*ortho/meta* ~1:1.8) ^3^
3	R = Me	53 (*ortho*), 13 (*meta*)	97 (*ortho*), 62 (*meta*) ^4^
4	R = CH_2_OEt	36 (*ortho*), 18 (*meta*)	94 (*ortho*), 100 (*meta*) ^4^
5	R = CH_2_OH	47 (*ortho*), 26 (*meta*)	21 (*ortho*), 42 (*meta*) ^4^

^1^ Data were obtained from reference [31]. ^2^ After 120 h of the reaction. ^3^ CH_3_ONa as a base. ^4^ Relative to the corresponding *ortho-* or *meta*-DA cycloadduct.

## Data Availability

Data sharing is not applicable.

## Abbreviations

2-MF	2-methylfuran
Ac	acetate
BAMF	2,5-bis(acetoxymethyl)furan
BHMF	2,5-bis(hydroxymethyl)furan
BMI	4,4’-bis(maleimido)diphenylmethane
BOC	*tert*-butyloxycarbonyl
Bn	benzyl
Bz	benzoyl
DA	Diels–Alder
DFT	density functional theory
DMF	dimethylformamide
DMSO	dimethyl sulfoxide
Emim	1-ethyl-3-methylimidazolium
EWG	electron-withdrawing group
FAM	furfuryl amine
FF	furfural
HMF	5-(hydroxymethyl)furfural
HOMO	highest occupied molecular orbital
IMDA	intermolecular Diels–Alder
LUMO	lowest unoccupied molecular orbital
MOF	metal organic framework
MPA	3-methylphthalic anhydride
N.d.	not determined
NMR	nuclear magnetic resonance
PEG	polyethylene glycol
rDA	retro-Diels–Alder
RT	room temperature
Tf	triflate
TFA	trifluoroacetic acid
THF	tetrahydrofuran
